# Content and Engagement Among Plastic Surgeons on Instagram

**DOI:** 10.1093/asjof/ojac096

**Published:** 2023-01-02

**Authors:** Kometh Thawanyarat, Chandler Hinson, Diego A Gomez, Mallory A Rowley, Yelissa Navarro, Chelsea M Venditto

**Affiliations:** Medical College of Georgia at Augusta University, Augusta, GA, USA; Frederick P. Whiddon College of Medicine, University of South Alabama, Mobile, AL, USA; Mayo Clinic Alix School of Medicine, Phoenix, AZ, USA; SUNY Upstate Medical University, Syracuse, NY, USA; Medical College of Georgia at Augusta University, Augusta, GA, USA; Medical College of Georgia at Augusta University, Augusta, GA, USA

## Abstract

**Background:**

Patients routinely use social media to locate providers, review before-and-after photographs, and discuss experiences, making it a powerful marketing tool for plastic surgeons. A few studies have systematically evaluated plastic surgery app content.

**Objectives:**

This study aims to analyze engagement levels and content posted by top plastic surgeon influencers on Instagram (Menlo Park, CA).

**Methods:**

The authors conducted a cross-sectional study in February 2022 to identify the top 10 global plastic surgeons on Instagram. Influencers were ranked based on the number of followers, and their latest 20 posts were analyzed. A total of 200 posts were categorized by 2 independent trainees as one of the following: marketing, education, personal, and miscellaneous. The number of likes was recorded as a proxy for engagement, and the average engagement for each category was calculated.

**Results:**

The top 10 influencers work primarily in private practice focusing on aesthetic procedures. Out of 200 categorized posts on Instagram, marketing posts had the greatest presence (64.5%), followed by personal (20%), miscellaneous (11%), and educational (4.5%). More still images were posted (56.5%) than videos (43.5%). The highest average engagement was for personal content (*P* = .005). No significant differences in engagement levels were found between photo and video content (*P* = .24).

**Conclusions:**

Although most content posted related to marketing efforts, many influencers were also using social media to post about their personal lives and promote their ancillary businesses. Although marketing content was the most common, engagement levels were the highest for personal and educational content, and no significant differences in engagement were found between videos and photos.

The growth of social media platforms has created a unique opportunity for plastic and reconstructive surgeons to directly interact with future patients. Social media platforms have significantly grown over the last decade with an ∼4.74 billion current social media users, equating to 59.3% of the total global population using at least 1 social media platform.^1^ The United States accounts for the greatest number of posts for plastic surgery. Interestingly, Seoul, South Korea, is the city with the greatest number of plastic surgery posts, beating out both Miami and Beverly Hills, demonstrating that the use of the platform for plastic surgery reaches a global scale.^2^ In the realm of plastic surgery, surgeons routinely use social media to advertise and demonstrate their surgical work and expertise to interested patients. Plastic surgeons can use these platforms to increase their practices’ website traffic. A recent online survey distributed to American Society of Plastic Surgery members found that 61.9% of respondents had an active professional social media account.^3^ A majority (79.4%) worked within aesthetics and cosmetic surgery. In the same online survey, respondents stated that the largest factors for creating and maintaining a social media platform were to “create an online presence” (83.0%), brand creation (45.6%), practice expansion (61.0%), and education (47.9%).^3^ Interestingly, although social media plays an important role in the advertisement of plastic surgery procedures to future patients, it is not associated with increased website traffic through the practice's webpage.^4^

From the patient perspective, social media provides an opportunity to “virtually” interact with the provider and see the provider's surgical results before making the commitment for an in-person consultation. Among the major platforms, Facebook (Menlo Park, CA) had the highest engagement among patients (50%), followed by Instagram (Menlo Park, CA) (30%).^5^ On these platforms, most patients engaged and viewed before-and-after photos of cosmetic surgical procedures they were interested in pursuing.^5^ Patients want to have a visual understanding of the procedures (final aesthetic results) in addition to understanding general information about the procedure, recovery times, etc. Many surgeons will post photos at various points in their patients’ recovery journey to provide a perspective of results over time to potential clients. Outside of outcomes and procedures, social media can be a platform to find a plastic surgeon. Because Instagram uses #hashtags as a means for navigating the platform, patients can search specific tags to view surgery-specific content. Plastic surgery providers will also use these same hashtags on their posts, allowing patients to directly connect with specific surgeons performing that specific procedure and the results of their work.^6^ This capability of Instagram allows it to be an ideal platform for plastic surgeons to directly market and connect with new and interested clients.

Although social media engagement between plastic surgeons and patients has become a growing field of academic interest, there is limited research into the type of posts and content which receive the largest amount of engagement from patients. Plastic surgeons often post a wide variety of content, including marketing, educational, and personal. By understanding patient engagement with these specific types of posts, plastic surgeons can tailor their social media marketing strategy to optimize patient views and patient engagement. Our study examines which Instagram posts receive the largest patient views and interactions.

## METHODS

### Data Collection

The 10 plastic surgeons with the most followers on Instagram were identified through a combination of Instagram and Google (Mountain View, CA) searches and ranked by follower count (Table 1). Board-certified plastic surgeons (or the equivalent certification in their country of practice) were included in the search.

**Table 1.** Top Plastic Surgery Instagram Influencers From February 2022

Number	Influencer	Username	Location	Followers	Posts
1	Michael Salzhauer	@therealdrmiami	Miami, FL	1,600,000	2024
2	Catherine Begovic	@beautybydrcat	Los Angeles, CA	1,200,000	2307
3	Laura Cala	@drlauracala	Bogota, Colombia	1,000,000	924
4	Terry Dubrow	@drdubrow	Los Angeles, CA	978,000	546
5	Tarick Smiley	@drsmiley	Los Angeles, CA	955,000	1152
6	Sheila Nazarian	@drsheilanazarian	Los Angeles, CA	822,000	2587
7	Tarik Husain	@dr.miamibeach	Miami, FL	815,000	306
8	Ashkan Ghavami	@drghavami	Los Angeles, CA	532,000	2534
9	Eugene Kim	@dreugenekim	Los Angeles, CA	373,000	1505
10	David Kim	@drdavidekim	Los Angeles, CA	267,000	1097

The 20 most recent posts up to February 2022 were captured for each surgeon's account. Two authors independently analyzed a total of 200 posts and categorized the content based on marketing, education, personal, and miscellaneous themes. The categorized posts were cross-referenced among the 2 authors to verify validity of content analysis. The definition for these categories can be found in Table 2. Like counts on each photo and view counts for each video were obtained as a proxy for audience engagement.

**Table 2.** Definition of Post Category

Category	Includes
Marketing	Practice endorsements and advertisements
Education	Explanation of procedure
Personal	Personal life: friends/family, accomplishments, political/advocacy, ancillary business promotion
Miscellaneous	Other

### Statistical Analysis

Two hundred posts were obtained from all 10 combined Instagram profiles. Total percentages and *like* averages were calculated among the categories. Analysis of variance test was run to determine any statistically significant difference among the categorical groups. Posts were then stratified by video vs photo within their categories and average engagement was obtained. *T*-test analysis was used to determine differences in video vs photo engagement among the categories.

## RESULTS

### Account Information

Information on the top 10 plastic surgeon accounts can be found in Table 1. These accounts are promoted as individual surgeons or a combination of surgeons and their practice. Of the 10 accounts, only 3 represented female surgeons and 7 represented male surgeons. The geographical distribution of the surgeons included 7 surgeons based in Los Angeles, CA, 2 surgeons based in Miami, FL, and 1 surgeon based in Bogotá, Colombia. The average follower count among these accounts was 650,000 followers (range of 267,000-1,600,00 followers). The average number of posts across the accounts was 1498 (range of 306-2587). Total likes among all 200 posts were collected for a total of 936,563 points of engagement.

### Content Analysis

**Figure 1.**
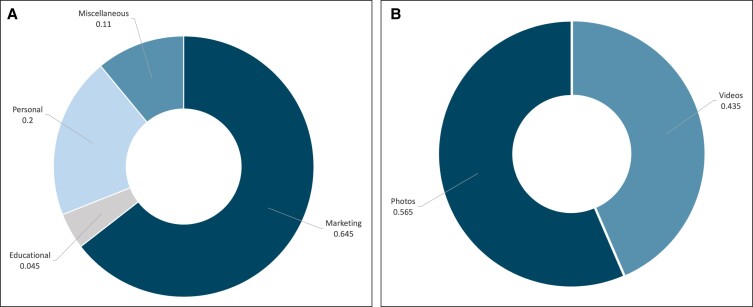
(A) The breakdown of post category among the 200 posts that were gathered for the plastic surgeon influencers. (B) The breakdown of post media type for the total content gathered: photos vs video format.

The percentage breakdown for the 200 categorized posts from the analyzed Instagram account can be found in Figure 1. Marketing (64.5%) content had the greatest presence, followed by Personal (20%), Miscellaneous (11%), and Educational (4.5%) content. Personal content was further classified into 4 categories: posts of friends and/or family (35%), ancillary businesses (35%), accomplishments (17.5%), and advocacy (12.5%). Still photo content (56.5%) was more prevalent than video content (43.5%) (Figure 1). There was a statistically significant difference in the average levels of engagement among the 4 content categories (Table 3). Personal posts had the highest average engagement, closely followed by educational posts, whereas miscellaneous and marketing posts had the lowest engagement (*P* = .005).

**Table 3.** Distribution of Instagram Content Engagement by Category

Category	Total posts	Total engagement	Mean engagement (range)
Marketing	129	482,322	3738.9 (50-25,436)
Personal	40	301,071	7526.8 (303-10,089)
Education	9	61,717	6857.4 (275-52,851)
Miscellaneous	22	91,453	4156.9 (43-17,726)
ANOVA *P*-value	.005	N/A	N/A

ANOVA, analysis of variance; N/A, not applicable.

**Figure 2.**
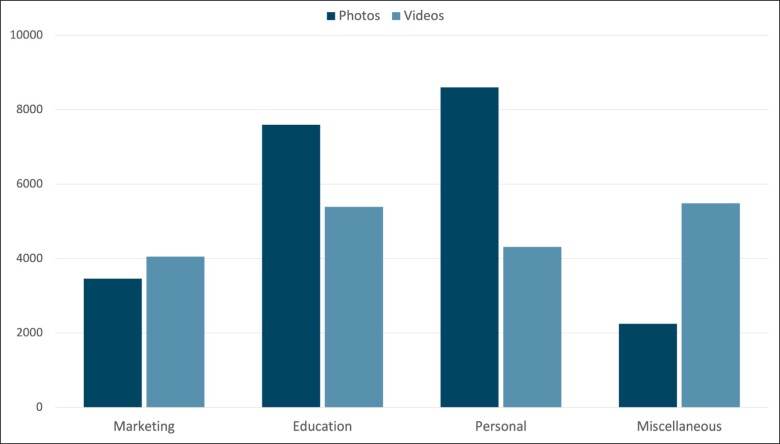
The amount of engagement on posts stratified by post category and media type within the category (video vs photo format).

Figure 2 shows the distribution of video compared to photo content among the Instagram accounts. When comparing engagement differences between photo and video content, there were no significant differences in engagement (*P* = .24) (Table 4). When analyzing photo vs video content within the categories of marketing, education, and personal, no statistically significant difference was seen (Table 4). However, the miscellaneous category did show greater engagement with video content compared to photo content (*P* = .023) (Table 4). On further analysis of the miscellaneous video content, 2 videos make up 43% of the engagement seen in the miscellaneous video category.

**Table 4.** Analysis of Photo Content Compared to Video Content Engagement per Category

Category		Photo	Video	*P* value
Total posts		113	87	N/A
Total engagement		558,848	377,715	N/A
Mean engagement		4989	4342	.47
Marketing	Total posts	68	61	N/A
	Total engagement	235,145	247,177	N/A
	Mean engagement	3458	4052	.43
Personal	Total posts	30	10	N/A
	Total engagement	257,962	43,109	N/A
	Mean engagement	8598.73	4311	.28
Educational	Total posts	6	3	N/A
	Total engagement	45,556	16,161	N/A
	Mean engagement	7593	5387	.42
Miscellaneous	Total posts	9	13	N/A
	Total engagement	20,185	71,268	N/A
	Mean engagement	2243	5482	>.05

N/A, not applicable.

## DISCUSSION

The utility of social media among plastic surgeons in practice has significantly increased in the last decade with a large proportion of social media presence led by private practice plastic surgeons.^3,7,8^ Among plastic surgeons, the value of social media has largely been accepted as an important platform to attract patients and educate the public—especially among new plastic surgeons.^9,10^ The connectivity of social media platforms like Instagram, Twitter (San Francisco, CA), and Facebook facilitates the growth of a surgeon's clientele base and is now considered a necessary step for the establishment of new graduating plastic surgeons.^11,12^ Through these platforms, surgeons are able to highlight their work, educate potential patients, and connect with others in their field. Interaction with these posts through likes and comments plays a large role in the surgeon's ability to reach their audience and keep them engaged on their content. Our study sought to identify which types of posts gained the most engagement among the top plastic surgeons on the social media platform—Instagram.

Our research found that among the top 10 plastic surgeons on Instagram with the greatest number of followers, marketing posts had the greatest presence but the lowest engagement. This aligns with previous studies that have shown that plastic surgeons are more likely to post marketing materials vs personal or educational materials.^13^ Personal posts had the highest average engagement followed by education, miscellaneous, and marketing content. This is in contradiction with other studies which have shown higher engagement with pre- and post-clinical photos vs personal and educational posts.^14^ When comparing photos to video content, there were no significant differences in engagement. These findings are in line with previous studies in the literature analyzing social media engagement for other specialties.^15^ Abbas et al similarly describe the content of posts published by orthopedic surgeons on various social media platforms and found that posts eliciting emotional responses and that solicited viewer responses were shown to have the greatest engagement.^15^ In a recent study, Wells et al reported similar findings among plastic surgeons on Instagram emphasizing higher follower engagement for personal and comedic videos compared to promotional videos when considering views as a proxy.^16^ Of note, as highlighted by previous studies, the category of “personal” includes posts that may convey both positive and negative emotions. Marketing posts, which may have a neutral stance with regards to eliciting emotion, show much less engagement in comparison.^8,15^ Our study demonstrates that the strategic addition of personal content may help increase engagement to expand the number of users that interact with their profile.

Social media has become a powerful tool to expand connectivity and increase practice exposure. The practice of plastic surgery, which previously relied on direct referrals from physicians or previous patients now uses platforms like Instagram and Facebook to increase public exposure.^10^ However, at its core, social media platforms were created for personalized connection among family, friends, and potential acquaintances.^17^ To this day, patients do not immediately utilize Instagram to find a provider of a certain service. As such, posts that present more personal content may have increased engagement and produce more traction among its audience compared to traditional marketing posts that tend to resemble advertisements. The increased engagement of personal posts by top plastic surgeons in the space provides a unique example of this phenomenon throughout our study.

Audience exposure and engagement in social media have been the focus of study for many researchers in the medical field.^9,11,15,17^ Plastic surgeons have pioneered the use of social media in practice given the consumer-driven nature of surgical procedures and the visually driven aesthetic outcomes throughout the specialty.^7^ Although the proper use of social media has been debated, no definitive guidelines are provided to direct specific usage of the platforms in terms of content to be shared and methods to increase interested patient exposure. Additionally, proper use commentary among plastic surgeons tends to focus on the sharing of patient information and photography that may be viewed as indecent or obscene by the general public.^3,7^ When scrolling through their feed, most individuals are not wanting to view graphic, post-surgical pictures, even if they demonstrate superior outcomes in comparison to other surgeons. Few studies have explored the content of social media posts and their effects on audience engagement.^5,15,16,18^ Dorfman et al study demonstrates that a strong social media following correlates with website ranking on the Google search results page.^12^ This finding emphasizes the importance of a social media presence and the value of strategic sharing of content. Personal posts, which provide less practice information, can improve a surgeons social media footprint which can increase exposure of marketing posts. Practice marketing posts, which receive less overall engagement on their own, may still provide information for potential new patients to the practice.^11,16^

Professionalism in social media has also been subject to discussion among plastic surgeons that use the platform for exposure. Debates on whether a surgeon should post their personal life and views is not clearly elucidated.^10,18^ Although patients younger than 35 prefer or are indifferent to viewing a plastic surgeon's personal life online, patients 35 or older do not prefer the additional exposure.^18^ As social media further permeates into everyday life, we expect this finding to further align toward favoring personal posts. More so as the younger public ages, they would express more acceptance toward their surgeons’ social media use in the medical setting. Recently, the main argument in the use of social media in plastic surgery has been in determining what may be deemed as professional or unprofessional. Professionalism in the specialty has been a central subject of debate led by guidance of policies like the ASPS Code of Ethics.^19^ Self-regulation by surgeons and researchers in the field also play a large role in determining what is deemed professional even as far as studies of the effect of a plastic surgeon's attire on patient–physician interaction.^20^ Yet the idea of professionalism may be affected by the context of what is being judged for medical professionals. One of the most recent examples of this involves the #MedBikini movement in which a now redacted article met negative reception in the medical community due to its judgmental basis centralized around a group of vascular surgeons taking part in activities that the authors perceived as unprofessional. Although the article itself does not provide any substance on professionalism, a large proportion of the responding commentary from physicians against their conclusion reflect a consensus in the community that showing your personal life is not unprofessional.^21^

Further studies show that there are nuances to sharing personal content. Overall, personal content provides a view to the prospective patient that their doctor can relate to them through their personal posts or interactions. Within cosmetic surgery, fostering a strong patient–physician relationship is crucial for long-term patients.^22^ Using personal posts can create and facilitate this interpersonal relationship, demonstrating its potential as a marketing strategy. An audience may not appreciate a post that feels like an advertisement, as we have seen in the results of this study. But if shared along with personal posts, the exposure to self-marketing posts may be more palatable for its viewers especially when the audience, or followers, are able to connect with the surgeons that are releasing the content. Current literature has attempted to put forth guidelines to help new surgeons navigate social media platforms and facilitate the exposure of their practice.^9,23–25^ The main consensus among these guidelines revolves around maintaining the central tenets that are embedded attributes of professionalism—promoting patient care that is safe, efficient, and effective; all the while maintaining patient comfort. The increased use of social media further underscores the need for an operational definition of professionalism while entering the era permeated with social media.

The lucrative return on investment for the use of social media in private practice is undeniable.^11^ Based on the top social media accounts highlighted in this study (Table 1), we see that there is much benefit for the utilization of social media platforms in the private practice setting. However, the role of social media in the academic setting remains in question. Many established academic plastic surgeons cited in the literature, especially senior academic surgeons, believe that the increased use of social media is leading to negative views on the specialty of plastic and reconstructive surgery.^9^ However, it is apparent that social media usage is increasing among residents in training and residency applicants to gain information on plastic surgery programs and their faculty, resources, and overall program setup.^9,26^ As such, the use of social media in academic plastic surgery has expanded to include not only plastic surgeons but residency programs as well.^8,27,28^ In the academic space, social media can be an asset for the expansion of the professional network among plastic surgeons, specialists, and the public. Our study demonstrates a similar trend with increased engagement among personal posts. In a study of posts by public residency program Instagram pages, Irwin et al show that the ideal posts to gain popularity involve content that includes residents operating and enjoying life outside of the hospital.^8^ The growing impact on social media in the academic setting has been considered an inevitable aspect of the specialty of plastic and reconstructive surgery. Integrating social media strategy and philosophy from top private plastic surgeons can be a helpful framework for forming a guideline for the use of platforms—like Instagram—for future discourse in academic plastic surgery and surgical training.

The cross-sectional nature of this study design creates limitations from the collection of data for a single point in time that may not account for ongoing changes in current trends that include changes in content creation and engagement count on the influencer's profile. Our analysis of likes as a proxy for engagement is working under the assumption that a like is a positive engagement metric despite the possibility that both positive and negative motives may drive this engagement. Live stories, which are available only for 24 h, were also not included in our analysis, which could help direct followers toward newly posted content. Additionally, no specific commentary was used in the engagement process, which could drive the Instagram algorithm to increased exposure of content. The subjective and complex nature of the Instagram algorithm may also promote content based on associated hashtags, initial engagement, and tagged accounts.

Despite these limitations, we were able to demonstrate that social media strategies, especially with Instagram, are vital marketing strategies. Future studies should focus on utilizing social media in all aspects of the specialty as a growing number of people begin to use it in their daily lives.

## CONCLUSIONS

Top plastic surgeons on Instagram obtain more interactions with their personal and educational posts, compared with marketing posts, despite the latter being more prevalent on their social media page. Marketing posts make up a large portion of content released, and although they have less engagement overall, they are more likely to share information about a plastic surgeon's practice. Although social media does not directly affect patient preference of plastic surgeons, showing personal posts can help patients relate to their surgeons, which has been shown to increase patient satisfaction, outcomes, and patient–physician rapport. These results represent a valuable tool in designing social media strategies for plastic surgeons. Increased social media presence is an important consideration for the field of plastic surgery as we attempt to expand connectivity among physicians, trainees, and the public in the new era of social media.
